# Prolonged Detection of Zika Virus in Vaginal Secretions and Whole Blood

**DOI:** 10.3201/eid2301.161394

**Published:** 2017-01

**Authors:** Kristy O. Murray, Rodion Gorchakov, Anna R. Carlson, Rebecca Berry, Lilin Lai, Muktha Natrajan, Melissa N. Garcia, Armando Correa, Shital M. Patel, Kjersti Aagaard, Mark J. Mulligan

**Affiliations:** Baylor College of Medicine, Houston, Texas, USA (K.O. Murray, R. Gorchakov, R. Berry, M.N. Garcia, A. Correa, S.M. Patel, K. Aagaard);; Texas Children’s Hospital, Houston (K.O. Murray, R. Gorchakov, R. Berry, M.N. Garcia, A. Correa, S.M. Patel, K. Aagaard);; Baylor St. Luke’s General Hospital, Houston (A.R. Carlson);; Emory University School of Medicine, Atlanta, GA, USA (L. Lai, M. Natrajan, M.J. Mulligan)

**Keywords:** Zika virus, viruses, infection, traveler, PCR, virus isolation, virus shedding, natural history, prolonged detection, saliva, urine, vaginal secretions, erythrocytes, whole blood, Honduras, United States

## Abstract

Infection with Zika virus is an emerging public health crisis. We observed prolonged detection of virus RNA in vaginal mucosal swab specimens and whole blood for a US traveler with acute Zika virus infection who had visited Honduras. These findings advance understanding of Zika virus infection and provide data for additional testing strategies.

Zika virus is a rapidly emerging mosquitoborne virus (*1*). In May 2015, Brazil reported autochthonous transmission of Zika virus (*2*). Over the course of 1 year, Zika virus spread to >50 countries and territories throughout the Americas (*3*). With the now confirmed link of Zika virus infection during pregnancy leading to fetal microcephaly (*4*) and reported cases transmitted by sexual contact (*5*), it is vital to understand the natural history of infection. We report an acute case of Zika virus infection in a traveler returning from Honduras to the United States and results from serial specimens collected for >11 weeks. These new data might serve as a potential guide for public health policy.

## The Study

This study was reviewed and approved by the Baylor College of Medicine Institutional Review Board (H-30533). A previously healthy, nonpregnant, 26 year-old non-Hispanic white woman returned to the United States from Tegucigalpa, Honduras, during mid-May 2016. Five days after her return (day 0), signs and symptoms consistent with Zika virus infection developed, beginning with rash and subsequent fever, headache, and conjunctivitis (Table). Fever and rash continued through day 5 and day 6, respectively. By day 15, desquamation was noted on the palms of both hands and soles of both feet. By day 17, all symptoms had resolved.

**Table:** Timeline of acute signs and symptoms and clinical progression/resolution for a 26-year-old woman infected with Zika virus who returned from Honduras to the United States

Day after illness onset	Signs and symptoms and clinical progression
0	Red, mottled, flat rash on stomach, back, and neck with pruritic progression over 24 h. Approximately 4 hours after first appearance of the rash, the patient became febrile (temperature 101.7°F), fatigued, and a headache developed.
1	Rash spread to the legs and upper arms, continuing to appear flat, mottled, and became pruritic. Fatigue persisted, along with headache with light sensitivity, myalgias (particularly in the back and shoulders), and nausea with anorexia. The patient reported that her eyes were painful to open, but upon examination, the eyes appeared normal with no redness or swelling.
3	The rash continued to progress to the entire body, but with decreasing pruritus and increasing papular appearance. Conjunctivitis and cervical adenopathy were noted on physician examination, and the patient reported increased myalgias and dysphagia, with development of painful vesicles throughout the oral mucosa.
5	Fever resolved
6	Rash and sore throat resolved
15	Desquamation was noted on palms of both hands and soles of both feet
17	Resolution of all signs and symptoms

Serial specimens were longitudinally collected for >11 weeks. The first specimens were collected on day 0, two hours after onset of rash and 2 h before development of fever. All remaining specimens were collected at 3, 8, 14, 21, 28, 35, 42, 53, 64, and 81 days after onset of illness. Specimens included serum, whole blood (EDTA anticoagulated), urine, saliva, and vaginal mucosa swabs. The patient was not menstruating when vaginal swab specimens were collected.

RNA was extracted from serum, whole blood, and urine samples by using the QIAamp MinElute Virus Spin Kit (QIAGEN, Valencia, CA, USA) according to the manufacturer’s instructions. Oral and vaginal mucosal swab specimens were collected by using the BBL CultureSwab Collection and Transport System (Becton Dickinson, Franklin Lakes, NJ, USA). Specimens were incubated in 250 μL of AL/carrier RNA lysis buffer for 10 min at room temperature; 200 μL of phosphate-buffered saline was added before RNA extraction. 

Eluted RNA from all samples was tested in a quantitative reverse transcription quantitative PCR (qRT-PCR) that included a TaqMan Fast Virus 1-Step Master Mix (ThermoFisher Scientific, Foster City, CA, USA) and a TaqMan ZIKV 1107 assay (*6*) with appropriate positive and negative controls. We detected Zika virus RNA in serum up to day 8 after onset of illness and in body fluids up to day 14; whole blood samples remained positive up to day 81 (Figure). Results of qRT-PCR of saliva were negative after day 8, and results for urine and vaginal swab specimens did not become negative until after day 14. We tested a day 0 serum sample for dengue virus and chikungunya virus RNA by using TaqMan assays (*7**,**8*); all results were negative.

**Figure:**
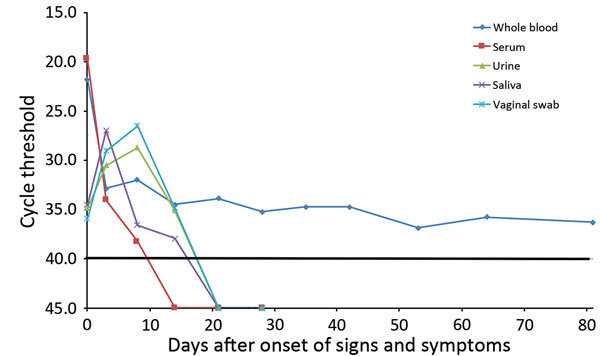
Quantitative reverse transcription PCR cycle threshold values over time (days after onset of illness) for whole blood, serum, urine, saliva, and vaginal mucosal swab specimens obtained from a 26-year-old woman infected with Zika virus who returned from Honduras to the United States. A cutoff value for a negative result was established at a cycle threshold of 40 (black horizontal line).

Virus isolations were performed for Vero cells in complete Dulbecco’s modiﬁed Eagle’s medium containing 10% heat-inactivated fetal bovine serum. Cells were infected with day 0 serum samples (or mock-infected with cell culture medium) and observed for cytopathic effects. Cell culture supernatants were sampled 13 days after cell culture infection, and RNA was extracted and tested for Zika virus RNA. Supernatant was collected on day 14, and viral titer was 8.5 × 10^5^ PFU/mL by plaque assay. Attempts to isolate virus from the day 64 erythrocyte fraction showed no evidence of cytopathic effects, and first and second passages were negative by qRT-PCR. Because the day 81 whole blood specimen was still positive by qRT-PCR, we used ficoll to separate peripheral blood mononuclear cells and erythrocytes and found that erythrocytes were the only fraction positive for Zika virus RNA. The partial sequence of the virus we isolated was submitted to GenBank under accession no. KX928077.

On day 8, plasma was evaluated by using an ELISA (*9*) to assess IgM and IgG binding to Zika virus envelope protein (Zika Virus Envelope Recombinant Protein, #R01635; Meridian Life Sciences, Memphis, TN, USA); positive results were obtained. Plasma-neutralizing antibodies against Zika virus were detected (50% focus reduction neutralization test titer 1:1,438), but neutralization of dengue virus serotypes 1–4 was not detected. These findings indicated a robust Zika virus–specific humoral response.

## Conclusions

Given recent concerns regarding the ongoing epidemic of Zika virus disease, there is an urgent need to document the natural history of infection and assess transmission risk through nonvector routes. We had the unique opportunity to prospectively monitor the clinical and virologic course of Zika virus infection in a patient starting on day 0.

We detected viral shedding in vaginal secretions up to day 14. Only 1 human study reported Zika virus RNA in cervical mucous up to day 11 after onset of signs and symptoms (*10*). These findings are supported by recent results for 2 animal models. Zika virus (Asian lineage strain) RNA was detected in vaginal swab specimens obtained on days 1 and 7 postinfection of nonpregnant female rhesus macaques (*11*). Zika virus replication was also detected in vaginal mucosa of mice (*12*).

We could not determine whether positive results by qRT-PCR indicated replicating virus. With the recent finding of possible female-to-male virus transmission (*5*), infectious virus might be present in the vaginal canal and could serve as a risk for sexual or intrapartum transmission.

We detected viral RNA in serum up to 8 days and in whole blood up to 81 days after onset of illness. Diagnosis of infection currently relies mostly on PCR detection of Zika virus in serum. With concerns for Zika virus infection during pregnancy, screening of whole blood might be more sensitive in identifying infected patients, particularly if an asymptomatic patient has traveled from an area where exposure is a concern, had high-risk sexual contact, or is convalescing and PCR for a serum sample would probably yield a negative result.

Our observation is further supported by another recent study that found whole blood samples positive for Zika virus by PCR up to 2 months postinfection (*13*). In our study, we confirmed that a positive result was attributed to the erythrocyte component of whole blood, similar to what has been found in studies of West Nile virus (*14**,**15*). One study found that West Nile virus adheres to erythrocytes and could infect Vero cells (*14*). Although we did not observe infectious virus associated with erythrocyte positivity for Zika virus at day 64, this finding is still of concern and requires further investigation. Because the last whole blood sample collected on day 81 was positive for Zika virus RNA, follow-up testing will continue to define the longevity of viremia in whole blood.

In conclusion, this case study advances understanding of the natural history of Zika virus. It provides new findings, including detection of Zika virus RNA in vaginal secretions up to day 14 and in erythrocytes up to day 81, the longest reported duration of detection in this sample type. A desquamating rash developed on the hands and feet of the patient, which we presume was related to her infection. To our knowledge, this finding has not been previously described.

Additional studies involving larger cohorts of acutely ill Zika virus–infected patients tested over a longer period would solidify our understanding of the natural history of infection, duration of viral detection, and clinical outcomes. These studies will enable further development of evidence-based policies regarding diagnosis and clinical management of Zika virus–infected patients.
